# The implications of dust ice nuclei effect on cloud top temperature in a complex mesoscale convective system

**DOI:** 10.1038/s41598-017-12681-0

**Published:** 2017-10-23

**Authors:** Rui Li, Xue Dong, Jingchao Guo, Yunfei Fu, Chun Zhao, Yu Wang, Qilong Min

**Affiliations:** 1 School of Earth and Space Science, University of Science and Technology of China, Hefei, China; 2 Key Laboratory of Aperture Array and Space Application, Thirty-eight Research Institute of China Electronic Technology Group Corporation, Hefei, China; 3 Atmospheric Science and Global Change Division, Pacific Northwest National Laboratory, Richland, WA USA; 4 Atmospheric Science Research Center, State University of New York, Albany, NY USA

## Abstract

Mineral dust is the most important natural source of atmospheric ice nuclei (IN) which may significantly mediate the properties of ice cloud through heterogeneous nucleation and lead to crucial impacts on hydrological and energy cycle. The potential dust IN effect on cloud top temperature (CTT) in a well-developed mesoscale convective system (MCS) was studied using both satellite observations and cloud resolving model (CRM) simulations. We combined satellite observations from passive spectrometer, active cloud radar, lidar, and wind field simulations from CRM to identify the place where ice cloud mixed with dust particles. For given ice water path, the CTT of dust-mixed cloud is warmer than that in relatively pristine cloud. The probability distribution function (PDF) of CTT for dust-mixed clouds shifted to the warmer end and showed two peaks at about −45 °C and −25 °C. The PDF for relatively pristine cloud only show one peak at −55 °C. Cloud simulations with different microphysical schemes agreed well with each other and showed better agreement with satellite observations in pristine clouds, but they showed large discrepancies in dust-mixed clouds. Some microphysical schemes failed to predict the warm peak of CTT related to heterogeneous ice formation.

## Introduction

Since 1940s^1^ or earlier, laboratory experiments have been showing mineral dust particles, with varied mineralogy and size, can initialize ice nucleation at warmer temperatures and lower super saturations comparing to those required by pure water freezing^2^. The insoluble surface of mineral dust particle provides the solid-liquid interface needed for stabilizing newly formed ice embryos and thus enhances the heterogeneous freezing process in super-cooled water. In real atmosphere, at temperatures significantly warmer than ~−37 °C the threshold of homogeneous freezing, multiple types of ice phase clouds (e.g. altocumulus, cirrus, deep convective clouds and the associated stratiform clouds) were observed with co-existence of mineral dust particles^3–8^. Such ice nuclei (IN) effect in deep convective clouds may further lead to significant enhancement of latent heat releasing, which in turn causes atmospheric dynamic response to it. The enhanced deep convective clouds by dust may have higher rain top height and cloud top height^9–11^. However, such microphysical-thermodynamic-combined effects highly depend on cloud evolution stages^12^ and cloud types^13–15^. For clouds with relatively weaker dynamics (e.g. those formed in the outflow of convection core) and small to medium cloud water path (less than 300 g/m^2^), the cloud droplets without enough uplifting force to reach homogeneous freezing temperatures may glaciate under dust-laden condition rather than in liquid phase at the same height in pristine condition. In addition, the depletion of water vapor and super cool water at warmer temperatures reduces the occurrence of homogeneous nucleation^16^ at colder temperatures. The net effects of the above processes were a warming of cloud top temperature (CTT) of such clouds, and the associated positive longwave radiation forcing can be as high as 16 w/m^2^ ^15^.

However, large uncertainties existed in satellite observational studies of such IN effect. First, the contacting of aerosol and cloud is questionable if only passive satellite observations without vertical resolution were used (i.e. it is unknown if the aerosol plume beneath the cloud top really contacted with the cloud body or just overlapped horizontally with it). Second, even if clouds and the aerosols did contact with each other, without the information of vertical updraft and horizontal wind, it is unknown whether or not aerosol particles really mixed with the cloud particles and were vertically transported to layers cold enough for ice formation. Since liquid water was necessary for ice formation even with enough IN^17^, the vertical transportation of dust and liquid cloud droplets from lower layer to upper layer is critical for the proposed IN effect. Thirdly, the CTT of ice clouds is controlled by both cloud dynamic effects and potential aerosol IN effects. It is almost impossible to untangle these two effects merely by using satellite observations^18,19^.

Current parameterizations of heterogeneous ice formation in cloud resolving model (CRM) such as Weather Research and Forecast (WRF) model are generally simple and do not link IN to aerosol properties. For example, the widely-used methods of Fletcher^20^, Cooper^21^, and Meyers^22^ relate IN number concentration to temperature and ice super-saturation, respectively. For any given temperature, those parameterized IN from different methods can encompass variations up to order of three in magnitude because of ignoring spatial and temporal variations in IN source particle type^23^. More observational evidences and model sensitivity studies are needed to quantify heterogeneous ice formation process with acceptable uncertainties.

In this study, we developed a satellite-CRM combined methodology to study the mineral dust IN effects on ice cloud top temperature and to assess the model sensitivity to different parameterizations. For possible case of cloud-dust interaction, we combined observations from satellite passive (e.g. the Moderate Resolution Imaging Spectroradiometer, MODIS on Aqua Satellite) and active sensors (e.g. Cloud-Aerosol Lidar with Orthogonal Polarization, CALIOP on CALIPSO satellite; Cloud Profiling Radar, CPR on CloudSat satellite) to obtain horizontal and vertical distribution of cloud and dust aerosol to ensure the contacting between them at first. Then we conducted a simulation of the case using cloud resolving model (WRF) and compared the simulations to satellite observations. After confirming the simulations can capture the main properties of the system, we utilized the simulated three-dimensional (3D) wind field combined with the satellite observed 3D distribution of cloud and aerosol to ensure the mixing of cloud and aerosol. Via doing this, we minimized the error in classifying cloud samples truly mixed with dust. In addition, we studied the sensitivity of WRF simulations of CTT to different microphysical parameterizations, initial conditions and cumulus parameterizations.

## Results

**Figure 1:**
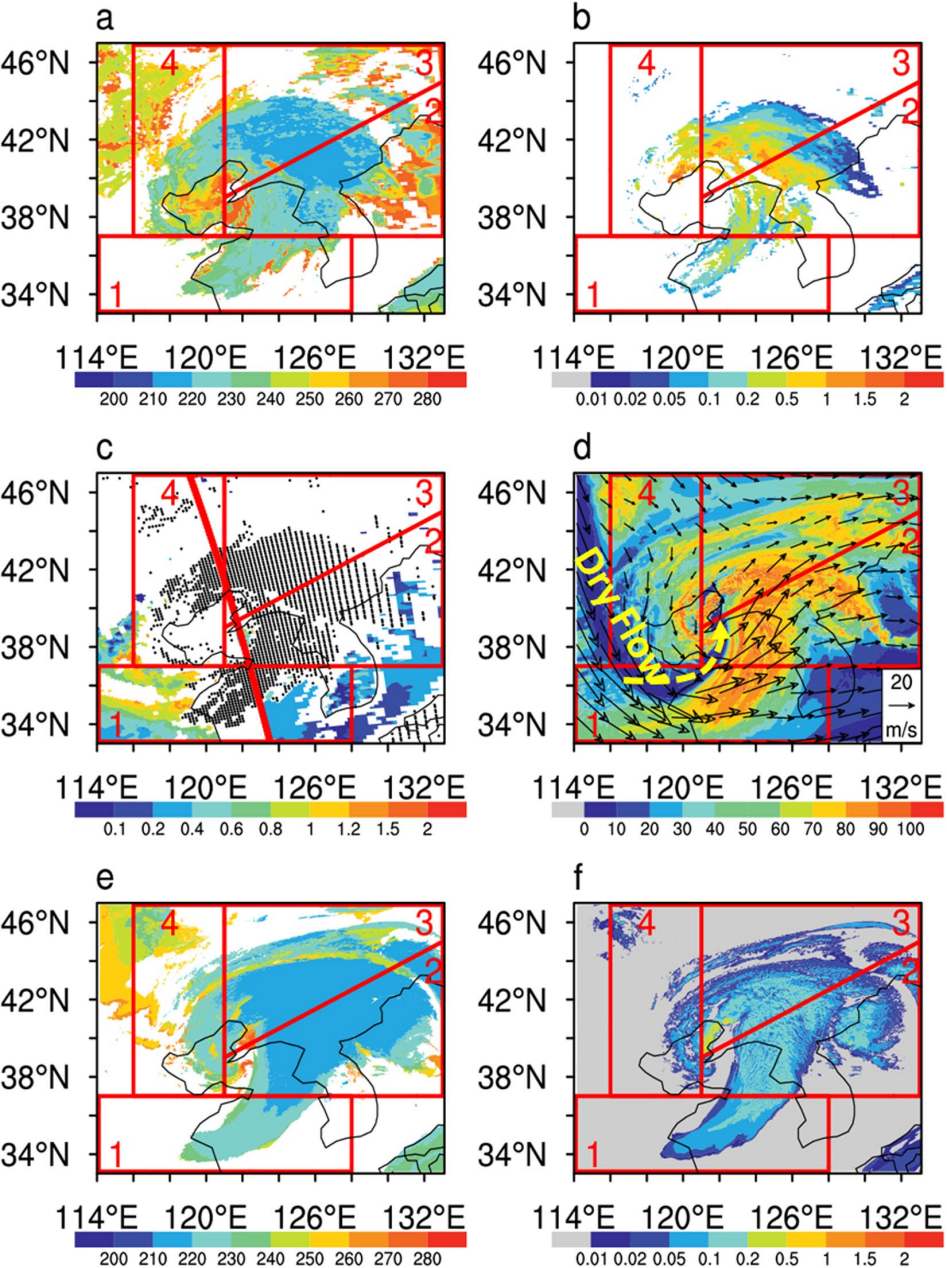
At 5:05 AM UTC on April 25, 2008: Horizontal distribution of (**a**) satellite observed cloud top temperature (K); (**b**) satellite observed cloud ice water path (kg/m^2^); (**c**) satellite observed coarse mode aerosol optical depth (black dots stand for locations of ice cloud, red curve stands for the nadir of the overpass of the A-train constellation) and (**d**) WRF-MOR model simulated horizontal wind field and relative humidity (%) at 500 hPa (with dry flow highlighted by the yellow arrow); (**e**) WRF-MOR model simulated cloud top temperature (K); (**f**) WRF-MOR model simulated cloud ice water path (kg/m^2^). Maps were created using NCAR Command Language (NCL, https://www.ncl.ucar.edu/) software version 6.2.0.

**Figure 2:**
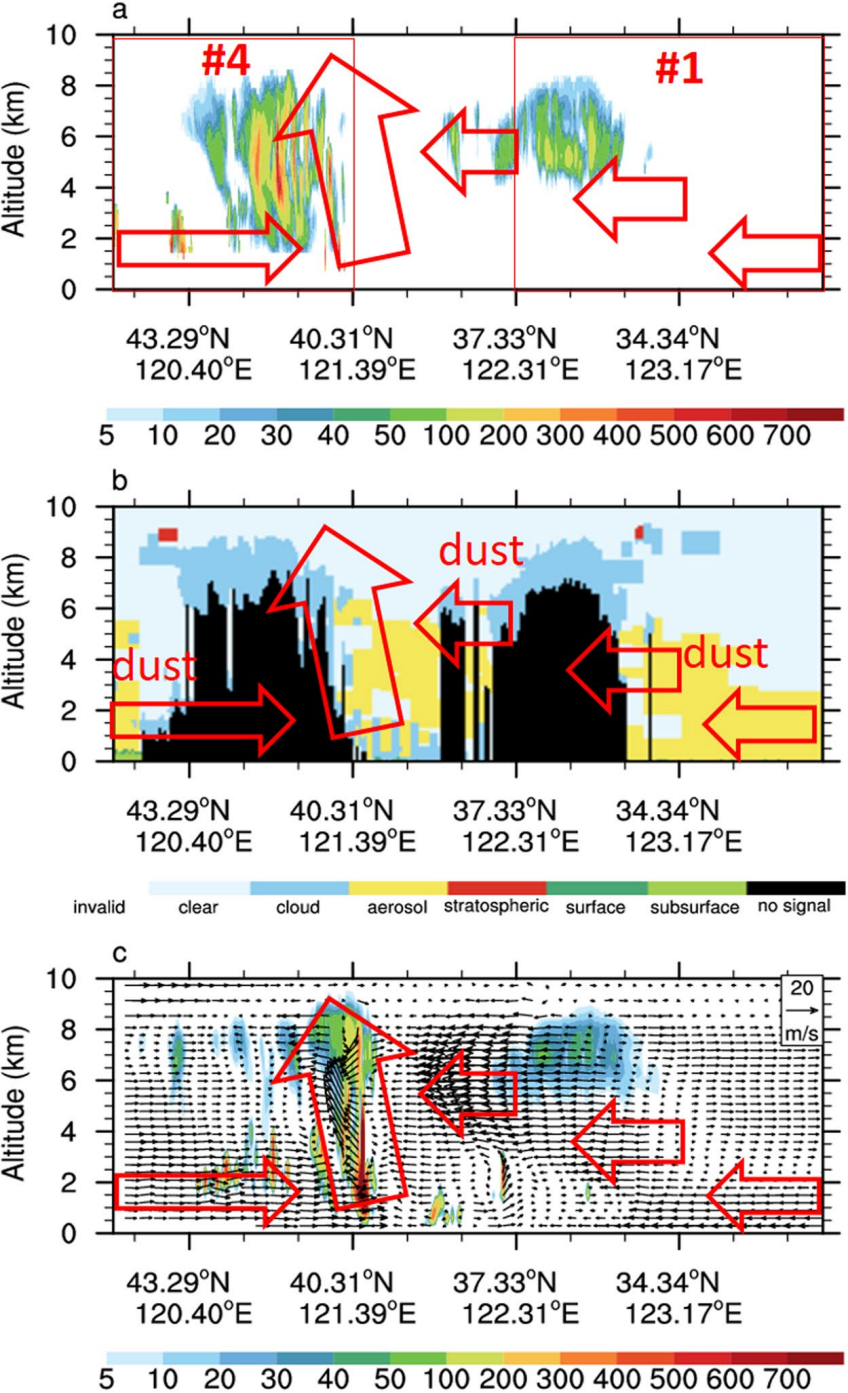
The vertical cross-section along the red curve in Fig. 1c for (**a**) cloud water content (mg/m^3^) derived from CloudSat/CPR; (**b**) cloud and aerosol detected by CALIPSO/CALIOP and (**c**) WRF-MOR model simulated cloud water content (mg/m^3^) and 3D wind field (updraft velocity was multiplied by a factor of 5). Red arrows represent the prevailing air motion at different locations and heights.

At 5:05 AM UTC on April 25, 2008, a typical case of dust-cloud interaction in Northeastern Asia was captured by Aqua, CALIPSO and CloudSat satellites in the A-train constellation^24–26^. High and thick deep convective clouds with widely spread ice clouds formed in this mid-latitude mesoscale convective system (MCS) with a clear comma structure indicating strong updraft and vorticity. The MODIS observed CTT (Fig. 1a) ranged from ~10 to −65 °C with cloud ice water path (CIWP) up to 2000 g/m^2^ in this system. Meanwhile, a massive dust storm originated from Gobi desert (refer to back trajectory analysis using NOAA HYSPLIT model in Fig. S1) invaded into this MCS following the large scale atmospheric circulation of typical cut-off low pressure pattern (Fig. S2). Strong spatial gradient of dust loading with greater coarse mode aerosol optical depth (AOD) to its western area than that to its eastern area provided a good opportunity to investigate the potential impacts of dust aerosol on cloud properties. Based on the horizontal distribution of cloud and AOD from MODIS (Fig. 2c), we divided the MCS into four sectors along anti-clockwise (marked from 1 to 4) direction including two relatively heavy dust loading (HD) sectors of No. 1 (AOD = 0.58) and 4(AOD = 0.78) and two relatively light dust loading (LD) sectors of No. 2 (AOD = 0.41) and 3(AOD = 0.34). Based on the horizontal wind derived from the dust-free WRF simulation using Morrison microphysical scheme^27^ (WRF-MOR; Fig. 1d), the dust stream directly inserted into the two HD sectors but had relatively weaker mixing with the two LD sectors at the moment of satellite overpass. Accompany with the dust particles was the dry air mass indicated by lower relative humidity (Fig. 1d), which fed the cloud body in HD sectors at low and mid layers.

The 3D distribution of clouds and aerosols along the red curve in Fig. 1c (mainly in selected Sector 1 and 4) were detected simultaneously by spaceborn radar CloudSat/CPR (Fig. 2a) and lidar CALIPSO/CALIOP (Fig. 2b). From observations of CPR who has good penetrating capability of cloud, we found the clouds in sector 4 formed in the outflow from a deep convective system with cloud bottom close to surface. The cloud in Sector 1 had much higher cloud bottom at about 4 km. Meanwhile, the identification of cloud and dust and their detailed locations were made by CALIOP. It showed the dust layer in Sector 1 and 4 extending from surface to 6 km altitudes.

The WRF-MOR simulation (Figs 1d–f and 2c) successfully captured the major features of this MCS, including the horizontal and vertical locations, the size of extent, the cloud water path, the CTT etc., comparing to the observations from CALIOP and CPR. We assumed the model simulated 3D wind field can be a reference to understand how the dust plume and the cloud were mixed. Those red arrows in Fig. 2 were added visually to indicate the main airflows at the interfaces between clouds and dust plumes and inside the convection core.

For clouds in Sector 4, the dust located at 0–3 km altitude to its northwest horizontally (Fig. 2) converged into the cloud bottom with wind speed at 10–15 m/s. In addition, the dust located at 4–6 km altitudes to its southeast also invaded into the cloud body at wind speed at 1–3 m/s. Both of these two “dust streams” met the strong updrafts (up to 8 m/s) in the convection core (centered at 121.39°E; 40.31°N) and were lifted up to high layer. It is very possible that cloud particles in sector 4 had strong mixing with dust particles based on the above observations and wind field analysis.

For clouds in Sector 1 without strong convection cores inside, the dust to its southwest extended from surface to high altitudes (up to 5 km) and horizontally conveyed to the cloud bottom layer (4.0–5.0 km) with wind speed around 7 m/s (Fig. 2c). Since the updraft inside the cloud layer was very weak in this sector, the mixing between cloud particles and dust particles in this sector should be weak despite the surrounding heavy dust loading (Fig. 1c). Therefore dust IN effect was expected to be much weaker in Sector 1 than that in Sector 4.

For given sector, it is not the ambient AOD that can solely determine the mixing between cloud particles and dust particles. We have to use the synthetic analysis of the 3D distribution of cloud, aerosol and wind field to find the real case of cloud-aerosol interaction. In Fig. 1a, CTTs in the sectors No. 2 and 3 were generally colder than those in the sectors No. 1 and 4. This was firstly related to the stronger updrafts there reflected by the greater CIWP (Fig. 1b), because CTTs were negatively correlated to CIWP^15^. On the other hand, the water vapor at low and mid layers is also important to determine the cloud top height^28^. The invasion of dry air mass (yellow arrow in Fig. 1d) may directly warm up the CTT in Sector 1 and 4 through thermodynamic mechanisms. In the following discussion, we only focus on ice clouds with CTT colder than −20 °C (to avoid the error of phase classification from MODIS) and CIWP less than 300 g/m^2^. These clouds covered most of our studying area.

**Figure 3:**
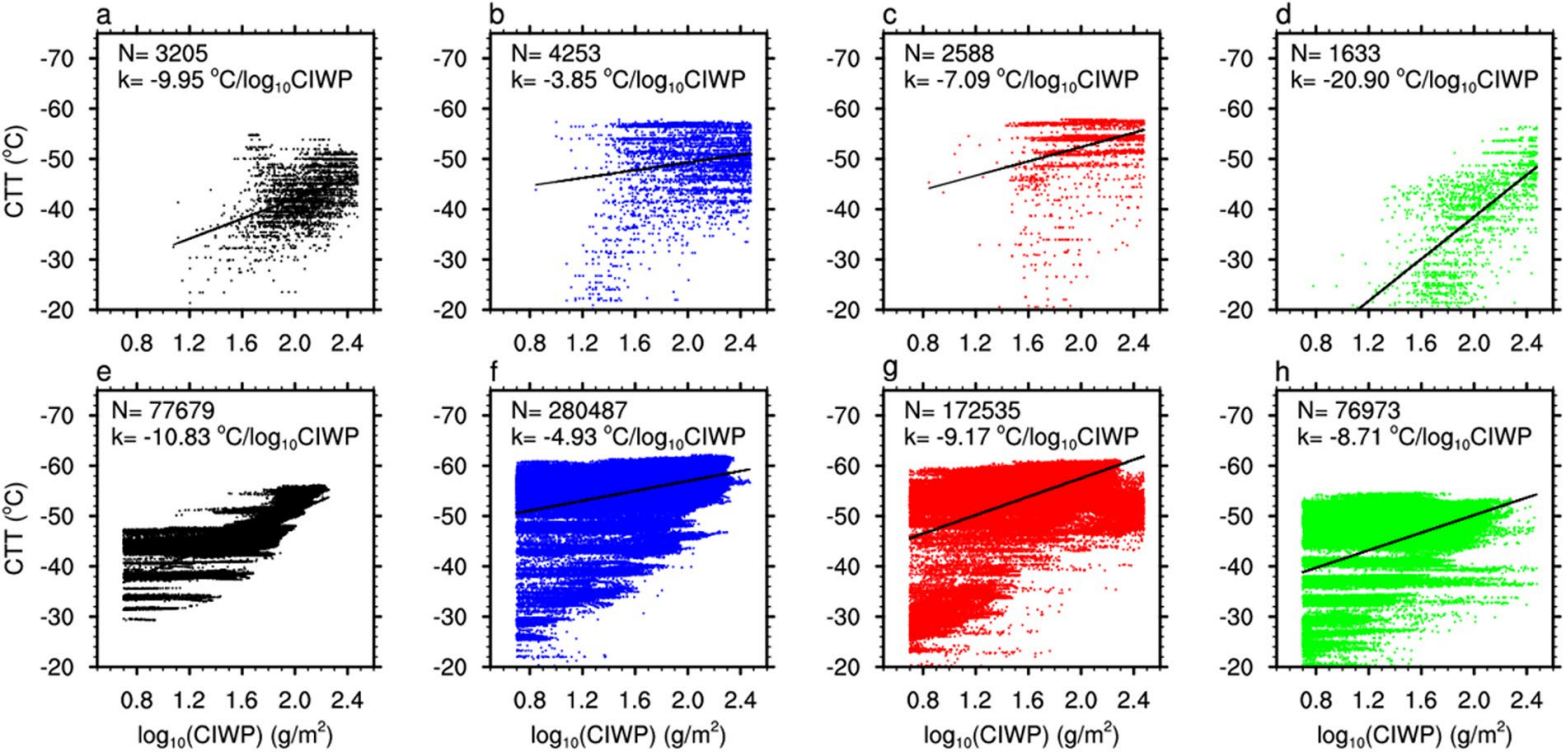
Scatter plots between CTT and cloud ice water path (CIWP, 5–300 g/m^2^, in logarithm scale) in selected Sector of 1, 2, 3, 4 (from left to right) based on satellite observations (upper row) and WRF-MOR simulations (lower row).

Although there were common negative correlations between CIWP and CTT based on satellite observations (Fig. 3), the linear regression slope of CTT against *log*_10_CIWP was steeper in sectors No. 1 and 4 (Fig. 3a,d) than that in sectors No. 2 and 3 (Fig. 3b,c). In Sector 4 with AOD 0.78, the slope was as high as 20.90 °C per unit *log*_10_CIWP which was about 5 times steeper than the value (3.85) in Sector 2 with AOD 0.34. This remarkable difference was mainly contributed by the ice clouds with CTT warmer than −38 °C in Sector 4. It indicated that heterogeneous ice nucleation process made strong contribution to ice formation in Sector 4 and caused warmer CTT for given CIWP.

In the WRF-MOR simulation (Fig. 3e–h), the slopes in Sectors of 1, 2 and 3 were generally close to those from the satellite observation. However, in sectors 4, the WRF-MOR simulated slope (8.71 °C per unit *log*_10_CIWP) was much smaller than that observed from the satellite (20.90 °C per unit *log*_**10**_ CIWP). This implies that WRF-MOR simulation can reasonably capture the CTT-CIWP relationship in light dust-laden conditions, but fail in heavy dust-laden conditions.

**Figure 4:**
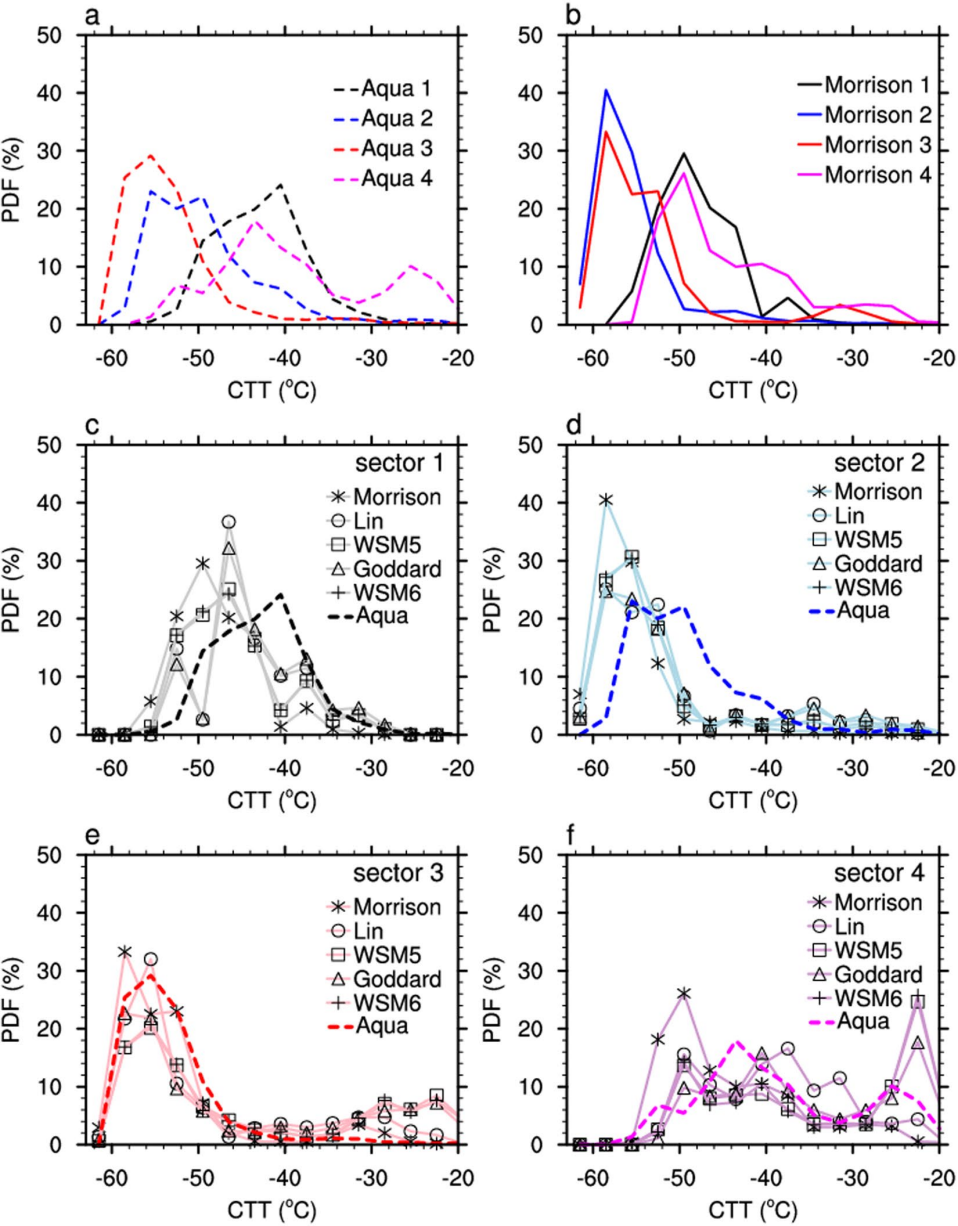
The Probability Distribution Functions (PDFs) of cloud top temperature (CTT) in the heavy dust-loading and light dust-loading sectors derived from (**a**) Aqua observations; (**b**) WRF simulations using Morrison scheme and (**c**–**f**) the comparison between satellite observation (dashed) against multiple WRF simulations with different microphysical assumptions in each selected sector of 1–4. Only ice clouds with ice water path 5–300 g/m^2^ were included.

From the point of view of CTT statistics in each sector, the satellite observed Probability Distribution Functions (PDF) of CTT in this case were divided into two groups. The PDFs in Sectors 2 and 3 (red and blue in Fig. 4a) showed peaks around −55.5 °C, which is definitely contributed by homogeneous ice formation. The PDFs in Sectors 1 and 4 (black and magenta in Fig. 4a) showed peaks located at much warmer temperatures of −45 to −40 °C. Remarkably, ice clouds in Sector 4 showed another “warm peak” of PDF around −25 °C, definitely contributed by heterogeneous ice formation. The mode of CTT in Sector 4 shifted about 12.2/17 °C (with/without counting the “warm peak” of PDF in sector 4) to the warmer end comparing to that in Sectors 2 and 3 (refer to Table S1).

**Figure 5:**
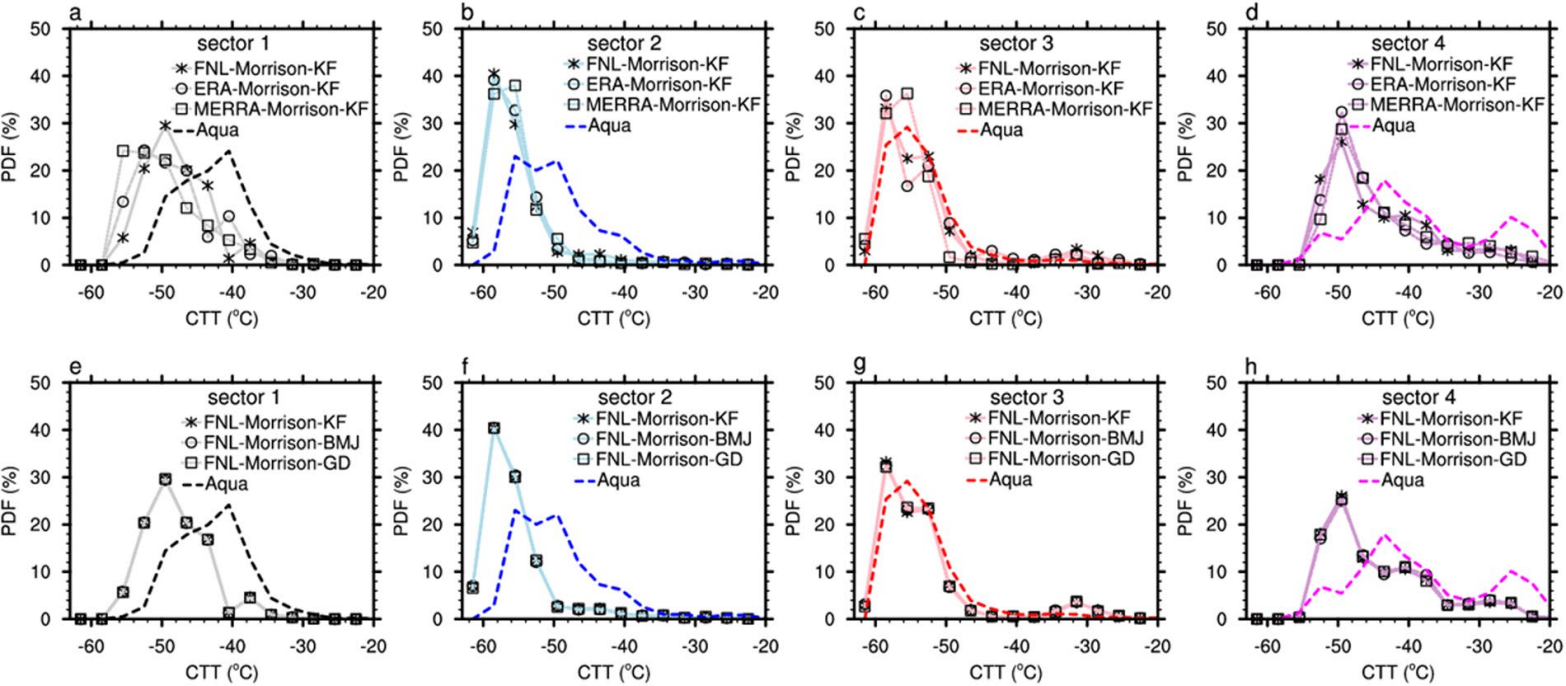
The Probability Distribution Functions (PDFs) of cloud top temperature (CTT) observed from Aqua and those simulated by WRF model with (**a**–**d**) different initial conditions and (**e**–**h**) with different cumulus parameterizations in selected sector of 1–4. Only ice clouds with ice water path 5–300 g/m^2^ were included.

Meanwhile, the WRF-MOR simulated PDFs of CTT (Fig. 4b) were also divided into two groups. The PDFs in Sector 2 and 3 were close to those in satellite observations with the mode of CTT around −58.5 °C (~3 °C colder than satellite observation). However, the simulated PDFs of CTT in Sector 1 and 4 were much colder than in satellite observations. Particularly, the observed CTT peaked around −25 °C in Sector 4 was completely missed by WRF-MOR simulation. Again, this is a strong evidence to show that the ice heterogenous parameterization in WRF-MOR can predict the PDF of CTT in relatively pristine ice clouds but cannot predict the behavior of ice clouds mixed with dust particles (i.e. those in Sector 4 in this case).

To investigate the sensitivity of CTT simulation to different microphysical parameterizations, we conducted four additional WRF simulations (WRF-Lin, WRF-WSM5, WRF-WSM6 and WRF-Goddard. Refer to Methods section.) of the same case shown in Fig. 4c–f. The results demonstrated that multiple WRF simulations matched the satellite observation the best in Sector 3 where the dust loading was the lowest. In sectors 1 and 2, the simulations of CTT at temperatures warmer than −37 °C (heterogeneous ice formation range) also matched the satellite observations well. Although the peaks of homogeneous ice formation in WRF simulations are about 5 °C colder than observations in sector 1 and 2, given the possible error of satellite retrieval (see later discussion), this difference is not significant. This confirmed that under light dust-laden condition, most ice formation parameterizations in WRF model can correctly represent the CTT of ice clouds with tolerable errors.

The largest discrepancies among multiple WRF simulations appeared in Sector 4. Compared to the satellite observations, at temperatures −20 to −40 °C, the WRF-MOR and WRF-Lin significantly underestimated the ice formation (very low PDF of CTT), while the WRF-WSM5, WRF-WSM6 and WRF-Goddard successfully simulated the peak of heterogeneous ice formation, but with some overestimations. This result implies that the ice formation parametrizations in WRF can lead to significant variations in the prediction of ice cloud properties at warm temperatures.

This MCS also was observed by another MODIS sensor on the Terra satellite about two hours in advance to the overpass of Aqua. However, we do not have associated observations from CloudSat and CALIPSO to identify the vertical distribution of cloud and aerosol at the overpass time of Terra. Following the same definition of Sectors 1 to 4, results (Fig. S4) from Terra observations were generally consistent with those mentioned above. The mode of CTT in the Sector 4 was about 12.5 °C warmer than that in the Sectors 2 and 3 based on Terra MODIS observations, while the WRF-MOR dust-free simulation showed the mode of CTT in Sector 4 was just 8 °C warmer than that in Sectors 2 and 3.

Both the satellite observations as well as the model results used in this study were obtained with high uncertainty. The cloud top temperature from MODIS was generated using the CO_2_ slicing algorithm. At satellite pixel level, the cloud top height (CTH) derived form MODIS for cirrus clouds can be 1–2 km lower (i.e 6–12 °C of CTT) than that from CALIPSO/CALIOP. For thick water clouds, the retrieved CTH from MODIS is within 0.25 km (~1.5 °C of CTT) of the Cloudsat/CPR backscatter determinations^29^. For our case, the ice clouds were related to a deep convective system, and therefore the instantaneous error of CTT form MODIS retrieval should be less than that for cirrus cloud but larger than that for water clouds.

The uncertainties in WRF modeling can come from different sources. The uncertainties related to microphysical parameterizations have been discussed already. To make assessment of WRF uncertainties from other sources, we further conducted 4 simulations under different initial conditions (two additional types) and cumulus parameterizations (two additional types, refer to method). At the resolution of 2 km, the standard deviation (σ) of WRF modeled CTT due to different initial conditions (e.g. NCEP FNL against ERA) is overall 3.58–9.26 °C (Fig. S6). The σ due to different schemes of cumulus parameterizations is 0.77–4.51 °C (Fig. S7). At this resolution, large deviations come from mismatch of individual cloud pixels predicted by different simulations.

If look at the statistics of CTT at each sector, the sensitivity of modeled CTT to different initial conditions led to some small variations in the CTT PDFs (Fig. 5a–d). None of the two additional simulations captured the warm peak at ~25 °C in sector 4 observed by Aqua MODIS. In addition, the sensitivity of modeled CTT to different cumulus parameterization is very small (Fig. 5e–h). This is due to the fact that cumulus parameterization is only applied to the outer one nested domain (with resolution of 18 km) so it actually only slightly affected the boundary condition of the inner domain (with resolution of 2 km). The associated results related to Terra MODIS observations are shown in Fig. S5.

## Discussions

The effects of dust aerosol acting as ice nuclei on cloud properties is still an open question. Although both ground-based and satellite-based observations showed some hints, large uncertainties existed in the data processing and methodology applied. In this study, we first designed a strict satellite data collocation methodology which combined active cloud radar, LIDAR and passive spectrometer observations to obtain the 3D distribution of cloud and dust. More important, we adopted 3D wind filed derived from cloud resolving modeling to clarify the transportation of aerosol, and to identify the place where the mixing between aerosol and cloud really occurred. We found in a mid-latitude mesoscale convective system, when ice cloud particles were mixed with dust, the linear regression slope of CTT against *log*_10_CIWP was about 5 times steeper than the value in relatively pristine ice clouds. In another word, for given ice water path, the CTT is warmer in dust-mixed ice clouds. And there were two peaks in the PDF of CTT in dust-mixed ice cloud, one is at about −45 °C, and the other is at about −25 °C, which definitely was contributed by heterogeneous nucleation. For pristine ice cloud, only one peak at −55 °C was observed.

The mechanism of heterogeneous ice formation remains one of the biggest challenges in cloud modeling. Through this study, the WRF simulation of ice cloud CTT is most sensitive to microphysical parametrizations, followed by the initial conditions. And cumulus parameterizations applied in the outer domain makes no effects on the simulation.

Overall, WRF simulations with different microphysical schemes agreed well with each other and showed better agreement with satellite observation in pristine ice clouds. However, for the ice cloud mixed with dust, the Morrison schemes failed to capture the slope of CTT-*log*_10_CIWP, and it completely missed the warm peak of CTT PDF at −25 °C. Meanwhile, the MSM5/6 and Goddard microphysical schemes successfully predicted this warm peak even with some overestimations. It demonstrated those microphysical parameterizations without taking into account the concentration of IN source aerosol (e.g. dust) may yield to large uncertainty in simulations and cannot explain the satellite observations.

## Methods

MODIS standard products of MYD/MOD04^30^ for aerosol optical depth and MYD/MOD06^31^ for cloud properties at resolution of 5 km were used in this study. The standard products 2B-CWC-RO of CPR cloud water content retrieval we used is based on Austin and Stephens^32^. The vertical mask features of dust and cloud retrieved from CALIOP/CALIPSO are based on the standard product of ^33^.

In this study, the Advance Research WRF model Version 3.4 was used to conduct cloud simulations without considering any dust aerosol effect. There were 55 vertical levels (250 m vertical resolution below 5 km altitude) and three nested horizontal domains with horizontal resolutions of 18, 6, and 2 km respectively in each simulation. The model was forced by the NCEP FNL (Final) Operational Global Analyses data with 1° by 1° spatial resolution and 6-hour temporal resolution. The outputs were saved at 30 minute intervals and the results from the first 24 hours were excluded to avoid spin-up errors. The Kain-Fritsch cumulus scheme^34^, the RRTM longwave radiation transfer scheme^35^, the Dudhia shortwave radiation transfer scheme^36^, and the YSU planetary boundary layer scheme^37^ were used in the simulation.

To investigate the sensitivity of WRF simulations to different microphysical parameterizations, we first used Morrison (WRF-MOR) and then used LIN^38^, WSM6^39^, WSM5^40^, and Goddard^41^ schemes to represent the modeling uncertainty with the standard deviation among them.

To investigate the sensitivity of WRF simulations to different initial conditions, we changed the data source of initial condition in the WRF-MOR simulation from NCEP FNL to ERA-Interim^42^ and MERRA^43^ reanalysis datasets. The associated simulations are marked as FNL-Morrison, ERA-Morrison and MERRA-Morrison, respectively.

To investigate the sensitivity of WRF simulations to different cumulus parameterizations, we changed the cumulus scheme in the WRF-MOR simulation from Kain-Fritsch scheme^34^ to Betts-Miller-Janjic scheme^44^ and Grell-Devenyi scheme^45^. The associated simulations are marked as Morrison-KF, Morrison-BMJ and Morrison-GD, respectively.

## Electronic supplementary material

Supplementary Information
