# Supplementary material for: The implications of dust ice nuclei effect on cloud top temperature in a complex mesoscale convective system
Source: Sci Rep. 2017 Oct 23;7:13826. doi: 10.1038/s41598-017-12681-0 (PMC5653839; doi:10.1038/s41598-017-12681-0)
Supplement: Supplementary file 1 — Supplementary Information [file 41598_2017_12681_MOESM1_ESM.pdf]

Supplementary Information

The implications of dust ice nuclei effect on cloud top temperature in a complex mesoscale convective system

Rui Li[1,+,*], Xue Dong[1,+], Jingchao Guo[1,2], Yunfei Fu[1], Chun Zhao[1,3] , Yu Wang[1,*], Qilong Min[4]

1. School of Earth and Space Science, University of Science and Technology of China, Hefei, China

2. Key Laboratory of Aperture Array and Space Application, Thirty-eight Research Institute of China Electronic Technology Group Corporation, Hefei, China

3. Atmospheric Science and Global Change Division, Pacific Northwest National Laboratory, Richland, WA, USA

4. Atmospheric Science Research Center, State University of New York, Albany, NY, USA

Table S1| Averaged coarse mode AOD and mode of CTT from satellite observations (CTT) and WRF-MOR simulations (CTT*) in selected sectors.

	Sector 1	Sector 2	Sector 3	Sector 4
AOD	0.58	0. 41	0.34	0.78
CTT (°C)	-40.5	-53.5	-55.5	-34.5[+]
CTT* (°C)	-49.5	-58.5	-58.5	-49.5

[+] The mode of CTT over the sector 4 is calculated as the arithmetic mean value of CTT with the double peaks (-44 °C and -25 °C) showing in Figure 5a.

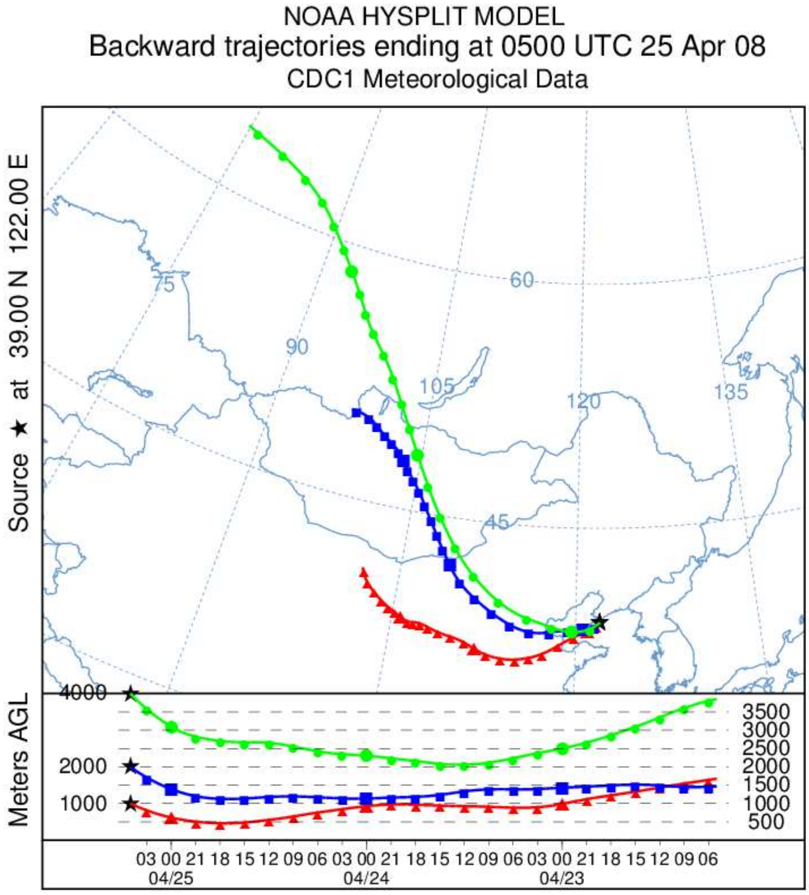

Figure S1| Back trajectory (72 hours) of air parcel in the center of cloud at 1000, 2000 and 4000m altitude derived from HYSPLIT model using CDC1 meteorology data. Plot was directly created by *the HYSPLIT transport and dispersion model and READY website (http://www.ready.noaa.gov)* of *NOAA Air Resources Laboratory (ARL)* .

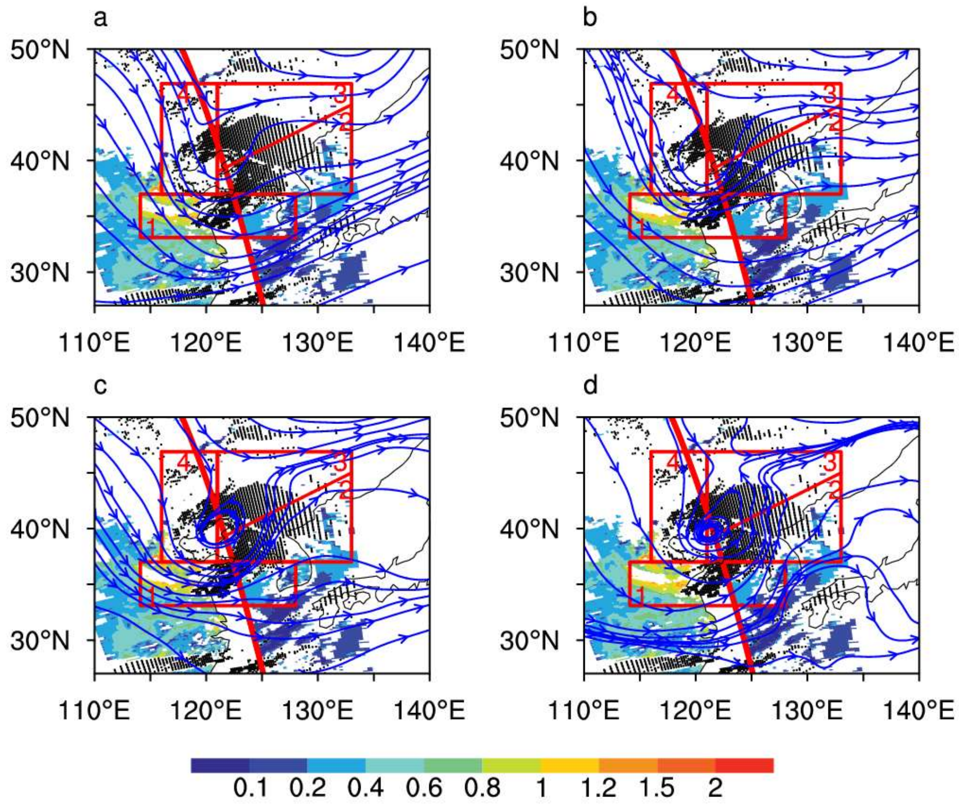

Figure S2|NCEP reanalysis of large scale circulation at (a) 300hpa, (b) 500hpa, (c) 700hpa and (d) 850hpa at UTC 6:00 AM Apr 25,2008. Overlapped were MODIS retrieved coarse mode AOD. Maps were created using NCAR Command Language (NCL, https://www.ncl.ucar.edu/) software version 6.2.0

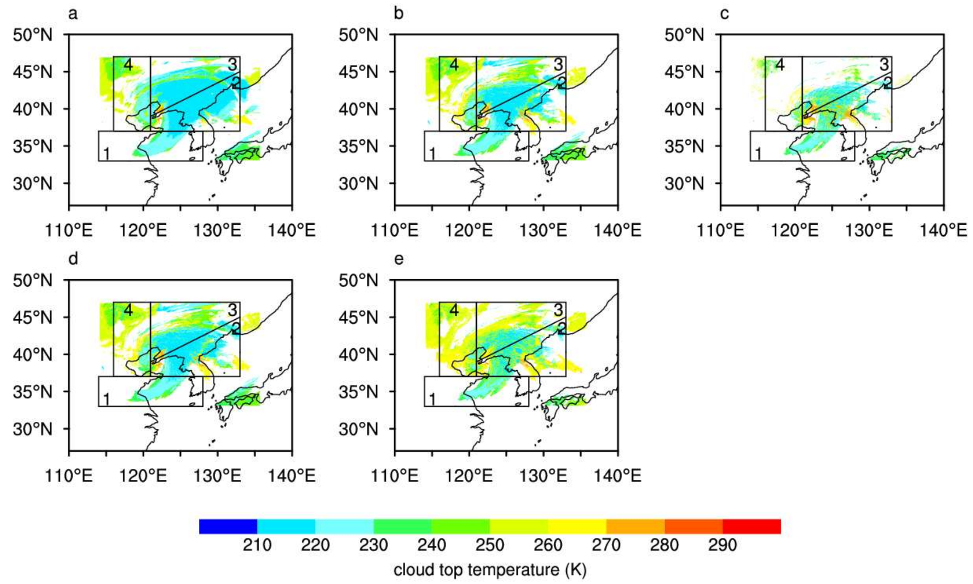

Figure S3| Cloud top temperature of ice clouds on 5:00 AM UTC at April 25, 2008 derived from dust-free WRF simulations with microphysical schemes of (a) Morrison; (b) WSM5; (c) LIN; (d) WSM6 and (e) Goddard. Maps were created using NCAR Command Language (NCL, https://www.ncl.ucar.edu/) software version 6.4.0.

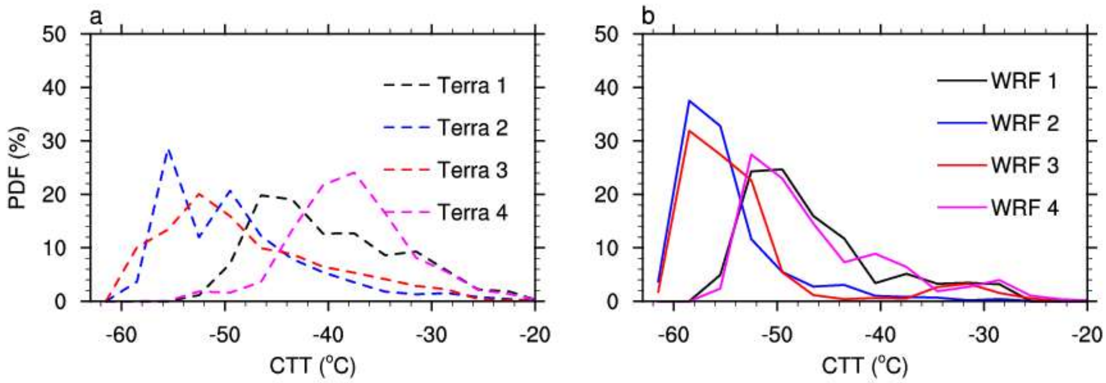

Figure S4| The Probability Distribution Functions (PDFs) of cloud top temperature (CTT) in the heavy dust-loading and light dust-loading sectors derived from (a) Terra observations ; (b) dust-free WRF simulations using Morrison scheme. Only ice clouds with ice water path 5-300 g/m^2 were included.

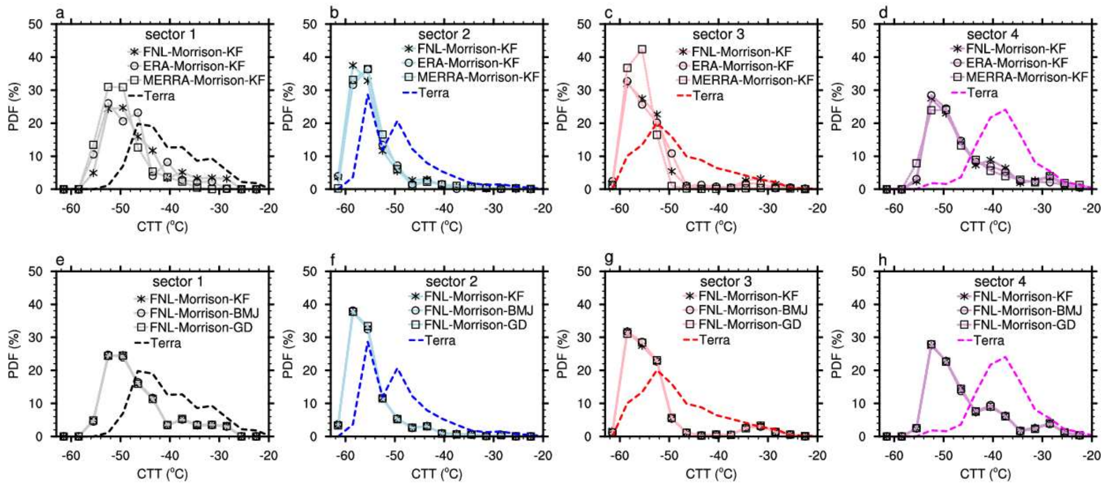

Figure S5| The Probability Distribution Functions (PDFs) of cloud top temperature (CTT) from Terra and those simulated by WRF model with (a-d) different initial conditions and (e-h) with different cumulus parameterizations in selected sector of 1-4. Only ice clouds with ice water path 5-300 g/m^2 were included.

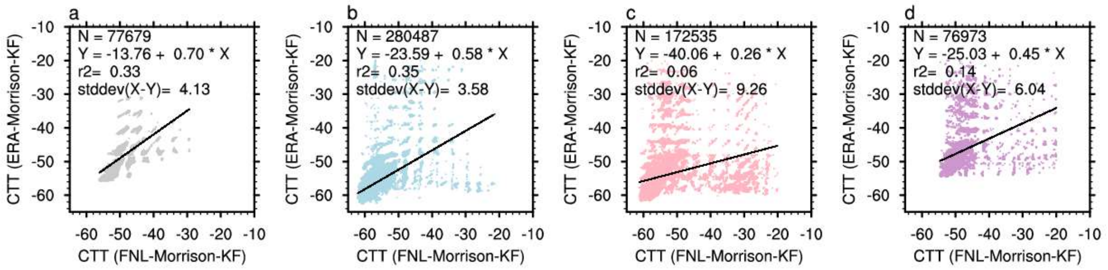

Figure S6| Scatterplots of WRF simulated CTT at 2km resolution using initial condition from NCEP FNL against those using initial condition of ERA-interin in selected sectors 1-4.

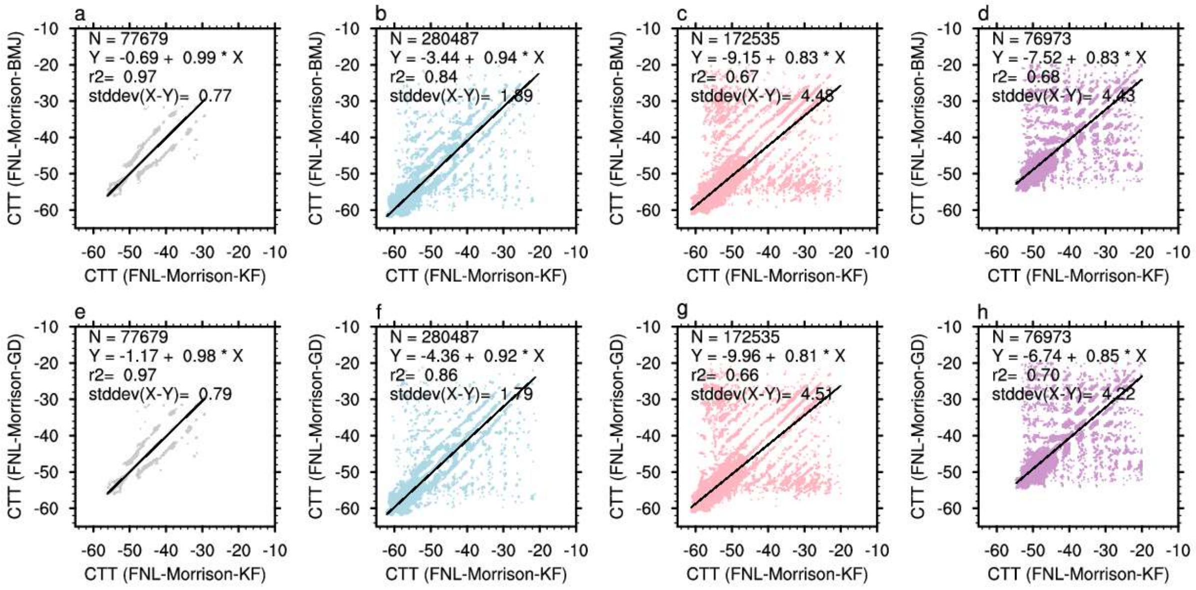

Figure S7| Scatterplots of WRF simulated CTT at 2km resolution using cumulus scheme of Kain-Fritsch against (a-d)those using cumulus scheme of Betts-Miller-Janjic (BMJ) and (e-h) cumulus scheme of Grell-Devenyi(GD), in selected sector 1-4.

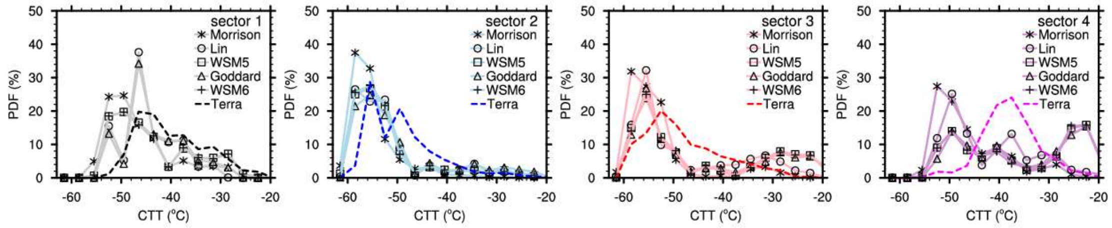

Figure S8| The Probability Distribution Functions (PDFs) of cloud top temperature (CTT) observed from TERRA and those simulated by WRF model with different microphysical schemes in selected sector of 1-4. Only ice clouds with ice water path 5-300 g/m^2 were included.
